# Coalescence-Induced Jumping of Multiple Condensate Droplets on Hierarchical Superhydrophobic Surfaces

**DOI:** 10.1038/srep18649

**Published:** 2016-01-04

**Authors:** Xuemei Chen, Ravi S. Patel, Justin A. Weibel, Suresh V. Garimella

**Affiliations:** 1 School of Mechanical Engineering and Birck Nanotechnology Center, Purdue University, West Lafayette, Indiana, 47907-2088, USA

## Abstract

Coalescence-induced jumping of condensate droplets from a superhydrophobic surface with hierarchical micro/nanoscale roughness is quantitatively characterized. Experimental observations show that the condensate droplet jumping is induced by coalescence of multiple droplets of different sizes, and that the coalesced droplet trajectories typically deviate from the surface normal. A depth-from-defocus image processing technique is developed to track the out-of-plane displacement of the jumping droplets, so as to accurately measure the droplet size and velocity. The results demonstrate that the highest jumping velocity is achieved when two droplets coalesce. The jumping velocity decreases gradually with an increase in the number of coalescing droplets, despite the greater potential surface energy released upon coalescence. A general theoretical model that accounts for viscous dissipation, surface adhesion, line tension, the initial droplet wetting states, and the number and sizes of the coalescing droplets is developed to explain the trends of droplet jumping velocity observed in the experiments.

Condensation heat transfer processes are essential to a range of applications, such as thermal management^1^, power generation^2^, water desalination^3^, and dew/fog harvesting^4^. Dropwise condensation can produce heat transfer coefficients that are an order of magnitude higher than filmwise condensation, if realized in industrial processes^5^. On superhydrophobic surfaces with nano-6,7,8,9,10 or micro/nano-structures^11^,^12^,^13^,^14^,^15^, condensate droplets can quickly be removed from the surfaces because of the minimal adhesion between droplets and the substrates, so that fresh spots on the surface are exposed for a continual process of nucleation and departure; this efficient dropwise condensation prevents the accumulation of a thick condensate liquid film on the substrate that would inhibit heat and mass transfer. Previous studies have demonstrated that condensate droplets can depart from superhydrophobic surfaces with a self-propelled out-of-plane jumping motion triggered by the surface energy release upon droplet coalescence^16^,^17^,^18^,^19^,^20^,^21^,^22^.

Coalescing droplets undergo a complex evolution in shape involving multiple dissipative factors that counteract the subsequent jumping motion. Several studies have attempted to explain the underlying physical mechanisms governing coalescence-induced droplet jumping based on a balance of system energies before and after coalescence, *i.e.*, surface energy, kinetic energy, and energies dissipated by viscous and surface-adhesion effects^16^,^17^,^18^,^19^,^20^,^21^,^22^. Boreyko and Chen^19^ were the first to report droplet jumping on superhydrophobic surfaces; they developed a simple capillary-inertial scaling to assess the droplet jumping velocity by assuming that all of the surface energy released is converted into kinetic energy. However, experimental droplet jumping velocities were significantly smaller than the predicted velocity, indicating the presence of other dissipative forces. Wang *et al.*^20^ included the additional effect of viscous dissipation caused by the flow and merging of droplets during coalescence on a superhydrophobic surface. The droplet jumping was shown to only occur for coalescence of droplets within a certain range of radii in which the surface energy dominates over the viscous dissipation energy. Subsequently, Lv *et al.*^21^ considered the additional surface adhesion-induced energy dissipation caused by contact angle hysteresis in their analysis, and showed that surface adhesion limits the critical size range for which droplets can jump, and reduces their velocity. In their follow-up work, the expression for adhesion-induced energy dissipation was modified for the coalescence of multiple equally size droplets^22^, and they showed that the jumping velocity increased with number of coalescing droplets. Tian *et al.*^23^ included dissipation energy due to the line tension at the three-phase contact line, and further demonstrated that minimizing solid-liquid adhesion is essential to realizing more efficient droplet self-propulsion from the surface. Several simulation studies considered the gravitational potential energy due to a change in the centroid height after droplet coalescence^24^,^25^,^26^.

All models reported in the literature are based on the assumption that *coalescence occurs for equally sized spherical droplets*; however, in reality, the bulk of condensate is removed from superhydrophobic surfaces via stochastic coalescence processes that occur for groups of droplets having different radii^11^,^12^,^13^,^14^,^15^. The detailed dynamics of jumping events involving multiple droplets of different sizes has not been explained by prevailing theories. Moreover, among all established models, none include the effect of surface micro/nano-structures on the droplet jumping behavior. For purposes of improving and validating available models, it is important to investigate the coalescence of multiple droplets of different sizes on hierarchical surfaces, which typically results in droplet jumping in a direction that deviates from the surface normal. An experimental technique that can map such three-dimensional droplet jumping trajectories is needed. Such experiments can clarify the droplet jumping mechanism and hence provide insights for designing suitable superhydrophobic surfaces for enhancing dropwise condensation.

In this work, we investigate the behavior of multiple-droplet coalescence-induced jumping during condensation on hierarchical superhydrophobic surfaces featuring nanostructured truncated microcones. On such surfaces, synergy between the micro- and nano-structures favors the formation of condensate droplets suspended on the inclined planes of neighboring cones, where the Laplace pressure assists the upward movement of the droplets^12^,^13^. A depth-from-defocus image processing technique is developed to track jumping droplets in three dimensions; this technique allows quantitative measurement of the jumping droplet size and out-of-plane displacement. The approach draws inspiration from vision systems observed in nature, such as that of the jumping spider which determines depth based on the variation in the level of chromatic abberation that results from the degree of image defocus^27^,^28^. As another example, human beings ascertain depth information in out-of-focus regions based on defocus blur^29^. The experimental results demonstrate that, in contrast to previously reported modeling predictions, the droplet jumping velocity decreases with increasing numbers of coalescing droplets, despite the greater surface energy released upon coalescence. A general theoretical model that takes into account the viscous dissipation, surface adhesion, line tension, droplet wetting state, and the number and size differences of the initial coalescing droplets is developed to explain the underlying jumping mechanisms that lead to the observed behavior.

## Results

### Surface morphology of the fabricated hierarchical superhydrophobic surface

**Figure 1:**
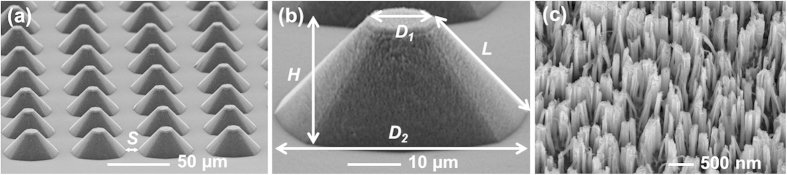
Scanning electron microscopy (SEM) images. (**a**) the hierarchical nanostructured truncated microcone surface, (**b**) an individual nanostructured truncated microcone, and (**c**) a high-magnification view of the nanostructures.

We fabricated hierarchical nanostructured truncated microcones on a silicon substrate using a two-step wet-etching process (see Methods Section for details). Briefly, the truncated microcone structures were first created using anisotropic wet-etching, following which nanostructures were etched across the entire surface using a metal-assisted chemical etching process. Figure 1 shows the SEM image of the fabricated surface. The top (*D*_*1*_) and base (*D*_*2*_) diameters of the truncated microcones are ~12 and ~45 μm, respectively. The edge-to-edge spacing between the bases of the truncated microcones (*S*) is ~10.5 μm, and the truncated microcone vertical height (*H*) and slant height (*L*) are ~23 μm and ~28 μm, respectively. The nanowires are ~150 nm in diameter (*D*_*n*_), ~1.5 μm in height (*H*_*n*_), and are spaced by ~350 nm (*S*_*n*_). The as-fabricated surface was silanized to render the surface superhydrophobic with a measured contact angle with water of 165° and a contact angle hysteresis less than 1°.

### Image processing technique to obtain depth from defocus

**Figure 2:**
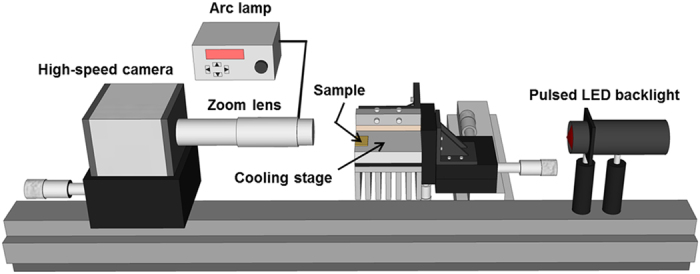
Schematic diagram of the experimental apparatus.

Condensation experiments were carried out in an ambient environment at a temperature of ~22 °C and relative humidity of ~35%. A versatile imaging platform was developed to perform microscale visualizations of the condensation process, as shown in Fig. 2 (see Methods Section for details). The as-fabricated superhydrophobic surface was placed on a temperature-controlled cooling stage. An arc lamp was used for continuous coaxial illumination and a pulsed-LED backlight was used to capture high quality freeze-frames of the jumping droplets in flight. Images were obtained using a high-speed camera and zooms lens.

**Figure 3:**
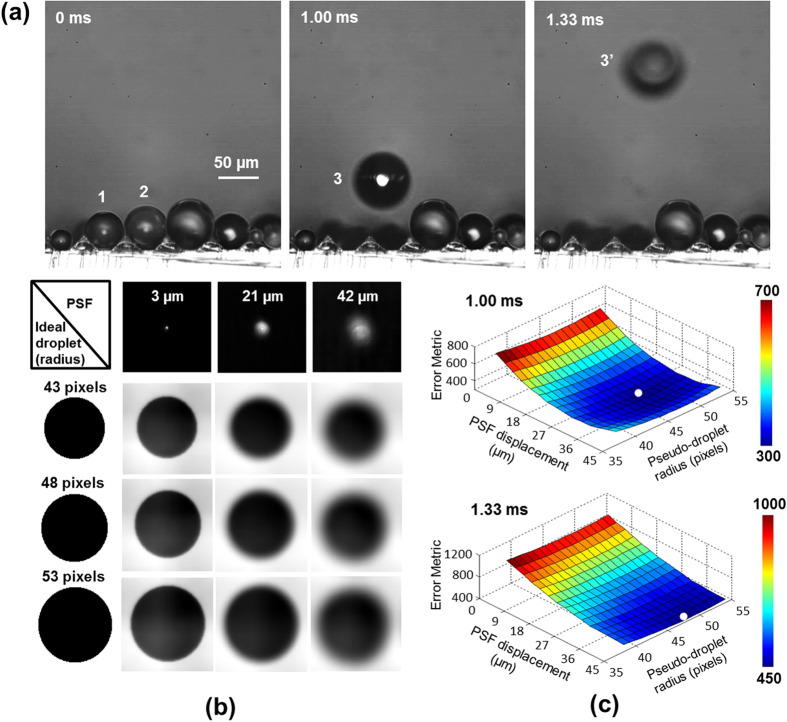
Image processing technique to obtain depth from defocus for the jumping droplets. (**a**) Images showing two droplets (marked 1 and 2) that coalesce into droplet 3, which jumps away from the surface, and is observed as the blurred droplet 3′ in a later frame. (**b**) The defocus space generation for the ideal pseudo-droplet image at varying sizes (left column), and varying point spread functions (PSF) obtained at different levels of defocus (top row). (**c**) Error surfaces of a jumping droplet at two consecutive frames (1.00 and 1.33 ms) as a function of pseudo-droplet size and level of defocus; the two white dots correspond to the minimums of the error surfaces.

Figure 3a shows representative snapshots of two droplets coalescing and jumping during the condensation process. When droplets marked 1 and 2 coalesce, the jumping trajectory of the coalesced droplet marked 3 clearly moves out of the focal plane and is observed as a blurred droplet denoted as 3′. A depth-from-defocus (DFD) image processing technique was developed and employed in order to obtain droplet position data that are resolved in all three dimensions for each frame obtained. Depth-from-defocus techniques rely on the quantification of the defocus blur at object boundaries as a means to determine object displacement out of the imaging plane^30^. This offers key advantages over stereoscopic approaches including avoidance of typical correspondence and occlusion problems^31^, as well as a more suitable and compact single-lens/single-camera imaging setup for observation of the microscale phenomena of interest in the present work.

The earliest implementations of DFD techniques were developed based on idealized simplifications regarding the imaging setup in order to correlate the degree of blur with the out-of-plane displacement^30^. The point spread function (PSF) of the lens, which governs the out-of-focus image alteration, was assumed in the early efforts to have a Gaussian shape and was applied to simplified lens-performance relations derived using ray optics. This contributed to a lower degree of measurement accuracy, especially under realistic conditions that include image noise^30^, non-circular lens apertures, and image distortions resulting from imperfections in lens manufacture^32^. The use of multiple frames obtained in static scenes at varying lens settings can increase technique robustness^33^, at the cost of reduced practical utility for dynamic applications such as the visualization of jumping droplets. For these reasons, it is desirable to develop a DFD approach that employs the real PSF in the characterization of defocus blur and also resolves depth data from a single image for the investigation of high-speed phenomena.

To obtain the real PSF for the imaging setup, a small backlit aperture (1 μm diameter) was visualized at varying levels of defocus ranging from 0 to 42 μm of out-of-plane translation in increments of 3 μm. After obtaining the PSF as a function of out-of-plane displacement, it is possible to refocus an out-of-focus image by deconvolving it with the PSF corresponding to the level of defocus in the original image^30^. However, while the presence of blurring makes it easy to visually determine when an image has been insufficiently refocused, it is challenging to determine when the image has been excessively refocused unless the imaging system has been modified to embed such information into the image via a coded aperture^30^. Thus, when using a lens with a circular aperture, such as that used in the present study, determining the PSF that corresponds to the exact displacement of an out-of-focus object is not a reliable procedure to obtain quantitative measurement data. Alternatively, images can be artificially defocused by convolving with the PSF at a given out-of-plane displacement. To circumvent the issues associated with refocusing actual images, a pseudo-droplet approach is proposed in the present work. The pseudo-droplet, a hypothetical phantom image of an ideal droplet in focus, is artificially defocused to match the characteristics of the real out-of-focus droplet observed experimentally. As the pseudo-droplet is defocused, the resulting blurring pattern at the droplet boundary is compared to that of the real droplet. This pseudo-droplet has a dark interior (pixel intensity = 0) and bright background (pixel intensity = 1), and is defocused by convolving the pseudo-droplet image with the various PSFs obtained experimentally in order to generate the defocus space, or the set of all images at varying levels of defocus. This procedure is repeated over a range of pseudo-droplet sizes in order to generate a look-up-table (LUT) against which experimental visualizations of real droplets are compared in order to simultaneously determine the out-of-plane displacement and size of the real droplets. An abbreviated sample of the LUT is shown in Fig. 3b, where several pseudo-droplet sizes are shown at different levels of defocus by convolving the ideal droplet image with the PSFs shown. The correct size and displacement of an experimentally observed jumping droplet are determined by the minimization of error between the defocus pattern of the real visualized droplet and the elements of the LUT, as calculated by summing the pixel-by-pixel absolute difference. Figure 3c shows the error surfaces of a jumping droplet at two instances (3 and 3′ at 1.00 ms and 1.33 ms) for a range of pseudo-droplet sizes and out-of-plane displacements. In this case, the minimum locations of the error surfaces for droplets 3 and 3′ correspond to the pseudo-droplet radius of 48 pixels (a radius of 38.4 μm) and the PSFs for out-of-plane displacements of 27 μm and 42 μm, respectively.

### Number of coalescing droplets in a jumping event

**Figure 4:**
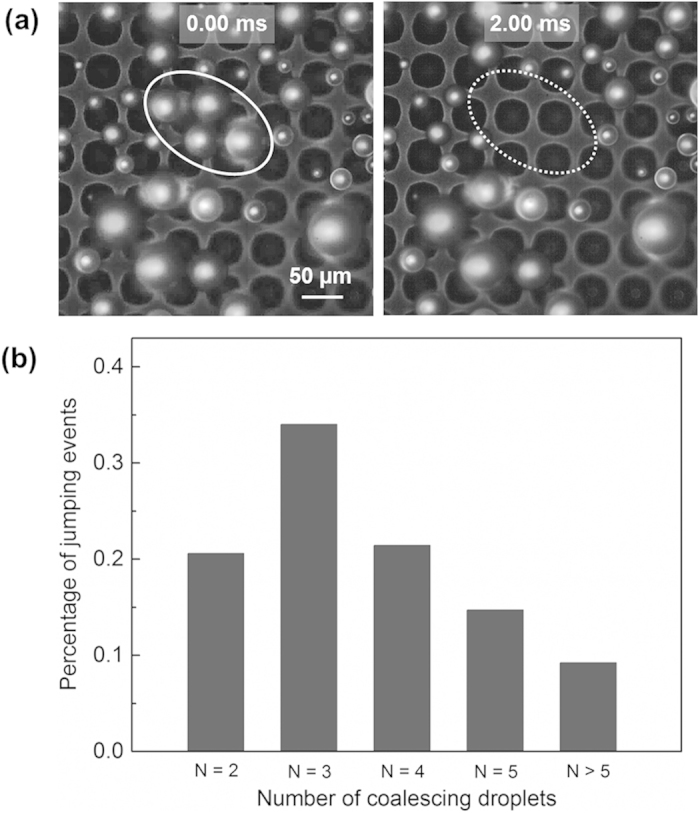
Determination of the distribution of droplet jumping events as viewed normal to the surface. (**a**) Selected condensation images showing moments before and after the departure of multiple droplets, as indicated by the solid and dotted ovals, respectively; (**b**) histogram showing the percentage of jumping events that result from different numbers of coalescing droplets.

The experiments show that the droplet jumping motion can be triggered by coalescence of two or more droplets. In order to determine the predominant jumping behavior that governs removal of condensate from the superhydrophobic surface, the frequency of jumping events is classified based on the number of participating coalescing droplets. The cooling stage is configured vertically to determine the number and size of participating droplets in coalescence-induced jumping events by viewing from a direction normal to the surface. Figure 4a shows an example view of the surface with 4 coalescing droplets before and after departure from the surface. Figure 4b shows the percentage of the jumping events versus the number of coalescing droplets for each over a sample size of 115 coalescence events viewed normally. While data and models are most commonly reported for the coalescence of two droplets in the literature, a majority (>79%) of jumping events occur upon the coalescence of multiple droplets (more than two and up to 10-plus); coalescence-induced jumping of three droplets is the most probable for our surfaces (~34% of the cases).

### Experimental droplet jumping velocities for different numbers of coalescing droplets

Coalescence-induced jumping events are viewed from the side to determine the droplet jumping velocity as a function of the number and size of coalescing droplets. However, coalescing droplets may mask one another when viewed by the camera from the side. To ensure an accurate count, we confirm that the total volume of visible coalescing droplets matches the volume of the droplet formed after coalescence. Using the DFD image processing technique, the sizes of the droplets are measured before (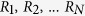) and after (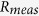) coalescence. The total volume of *N* coalescing droplets is calculated assuming they have spherical-cap shapes (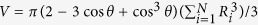 ) with a contact angle *θ* ~165°. The anticipated radius of the coalesced droplet (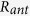) is calculated assuming the droplets should coalesce into a single sphere of volume 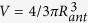. This is compared to the measured radius (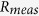) of the coalesced droplet in Figure S1 (Supplementary Material). Cases in which the difference is larger than 10% are discarded, and of the 65 total remaining cases, 50 are selected to achieve the same relative distribution of jumping events as for the entire dataset shown in Fig. 4b (with priority given to cases with the minimum discrepancy).

**Figure 5:**
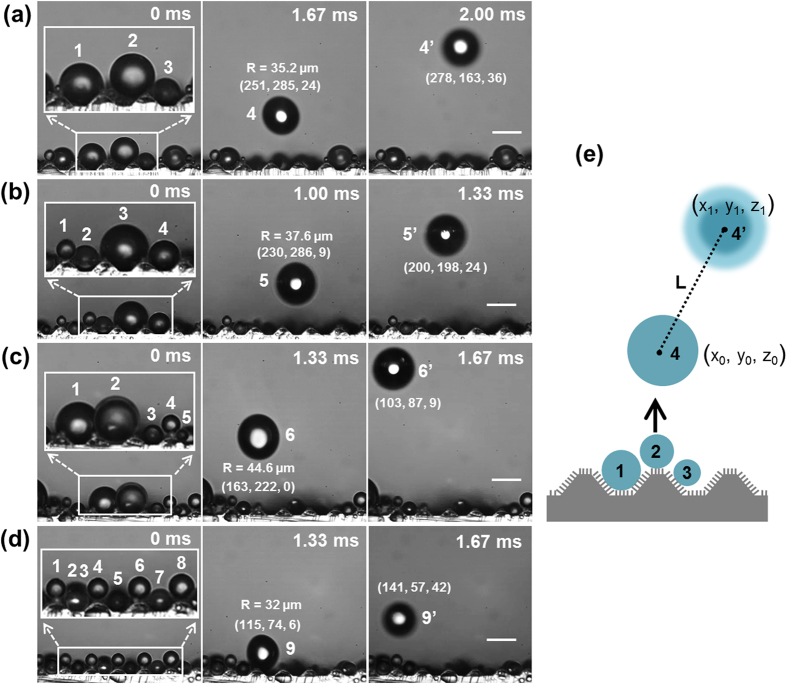
Representative cases of coalescence-induced droplet jumping events on the hierarchical superhydrophobic surfaces. Droplet jumping is induced by the coalescence of (**a-d**) three, four, five and eight droplets (marked with numbers in the inset images), respectively. The radius and coordinates (in microns) of the jumping droplets are obtained using the DFD technique; the origin is in the top-left corner of the image. The scale bars are 50 μm. (**e**) Schematic drawing showing the calculation of the distance between two consecutive jumping droplets, expressed as: 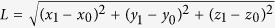.

**Figure 6:**
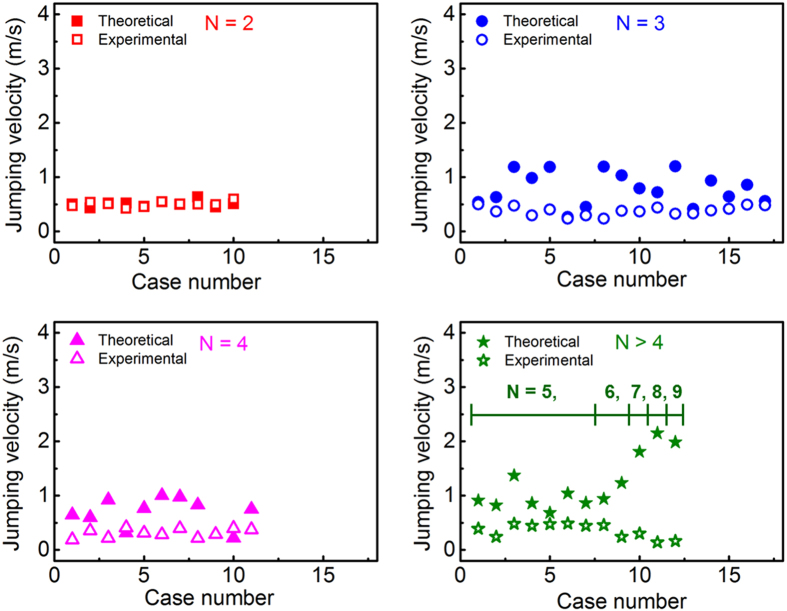
Comparisons between experimental droplet jumping velocities and theoretical predictions (using our modified model) as a function of the number of coalescing droplets.

Figure 5a–d shows representative cases of droplet jumping triggered by the coalescence of three, four, five and eight droplets. The radius of the jumping droplets (*R*) and the out-of-plane displacement between frames (*z*-coordinate) are obtained using the DFD technique. The droplet jumping velocity can be calculated based on the time interval and distance between two consecutive jumping droplets (Fig. 5e). Based on the in-plane and out-of-plane imaging resolution, the uncertainty in a single measurement of the velocity magnitude is ±0.019 m/s. The experimental droplet jumping velocities as a function of the number of coalescing droplets *N* are shown as the open symbols in Fig. 6. The maximum velocity is observed for two coalescing droplets, and is always lower for groups of multiple coalescing droplets. The average velocities (± the standard deviation) for *N* = 2, 3, and 4 droplets coalescing and jumping are 0.5 ± 0.046 m/s, 0.37 ± 0.082 m/s, and 0.31 ± 0.076 m/s, respectively. However, when more than four droplets coalesce, it may be inappropriate to average among the corresponding coalescence cases because the probability of jumping events for N > 4 is small (~28%), and there is not a statistically significant trend in the droplet jumping velocity with the number of coalescing droplets. This observation contradicts prevailing theoretical models that only include the energy dissipation caused by viscosity and contact angle hysteresis, and predict that the jumping velocity should increase significantly with the number of coalescing droplets^22^.

## Discussion

### Theoretical modelling of droplet jumping velocity

**Figure 7:**
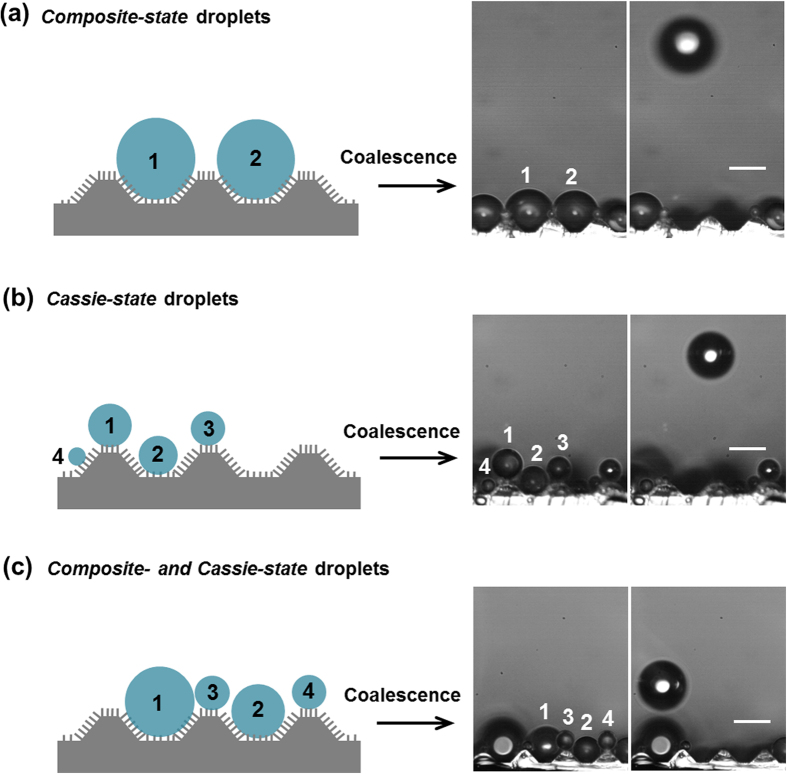
Wetting states and coalescence-induced jumping of condensate droplets. (left) Schematic drawing of the possible wetting states of condensate droplets on the hierarchical superhydrophobic surface, and (right) droplet jumping that is induced by the coalescence of (**a**) only composite-, (**b**) only Cassie-, and (**c**) both composite- and Cassie-state droplets. The scale bars on the images on the right are 50 μm.

Recent theoretical models that quantitatively predict jumping velocities are based on the balance between the released surface energy 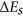 (Δ*E*_*s*_ = *E*_*s*_−*E*_*s,aft*_, where 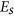 and 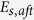 are the surface energy before and after droplet coalescence) and dissipative forces, *viz.*, viscous dissipation energy 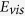, interfacial adhesion-induced dissipation energy 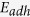, and kinetic energy 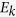. The models have been proposed for predicting the droplet jumping velocity from the coalescence of two equally sized droplets on superhydrophobic surfaces with only nanostructures^16^,^17^,^18^,^19^,^20^,^21^,^24^,^25^. Although these approaches can accurately describe the influence of droplet radius on the coalescence-induced jumping velocity, they do not consider the effects of droplet size differences (Supplementary Figure S2), initial droplet wetting states, and the presence of microstructures on the substrate. Unlike nanostructured superhydrophobic surfaces, the condensate droplets on the hierarchical superhydrophobic surface may reside in a composite state (see droplets 1 and 2 in Fig. 7a), where the droplet is larger than the microstructure and sits in contact with four confining truncated microcones. Alternatively, when the droplets are smaller than the microstructures, they sit in a Cassie state on the nanowires only (see droplets 1 through 4 in Fig. 7b). Droplet coalescence can occur for various combinations of composite and Cassie-state droplets on the hierarchical surfaces (Fig. 7c). Thus, it is difficult to formulate a universal energy equation to describe all possible coalescence conditions. In the following section, we introduce energy terms to accommodate prediction of coalescence between droplets in multiple wetting states.

We assume the Cassie-state droplets are of a spherical-cap shape sitting on the nanowires with a contact angle *θ* of ~165°, and the composite-state droplets fully contact the sidewalls of the truncated microcone and the interstitial valleys between, and therefore can be treated as a Cassie-state droplet sitting over a larger area of nanowires. The ratio of the contact area underneath the composite-state droplet to that of the Cassie-state droplets is the surface roughness of the truncated microcones (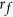), which is expressed as 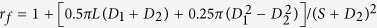. The surface energy of a single Cassie-state droplet is derived in Ref. 34; the surface energies of multiple droplets before coalescence 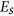 is equal to the sum of the surface energy of all individual coalescing droplets. Thus, the equations for 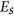 of multiple Cassie-state droplets (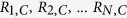) and multiple composite-state droplets (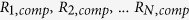) are respectively expressed as follows:


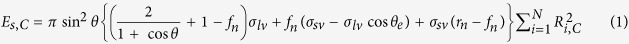



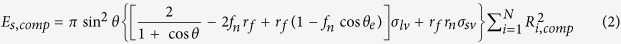


where 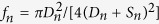 and 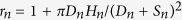 are the solid fraction and surface roughness of nanowires, respectively; 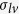, 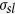 and 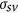 are the liquid-vapor, solid-liquid, and solid-vapor surface tensions, respectively; and 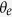 ~ 115° is the equilibrium contact angle on the silane-coated flat surface. When Cassie-state droplets coalesce with composite-state droplets, the surface energies in equations (1) and (2) can be summed together.

The viscous energy dissipated by the deformation of each droplet during coalescence is given by^20^,^35^:


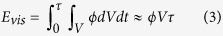


where 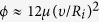 is the dissipation per unit mass of the fluid, *μ* = 1.01 mPa s is the dynamic viscosity of water, 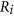 is the radius of a single coalescing droplet, 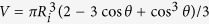 is the volume of the droplet, 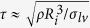 is the time required for coalescence, and *ρ =* 998.2 kg/m^3^ is the density of water. The capillary pressure inside the droplet 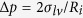 drives the droplet to coalesce in the horizontal direction, and the average merging velocity 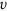 during the time 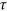 can be estimated as 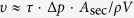
36, where 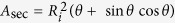 is the cross-section of the droplet.

The viscous dissipation is the sum of the dissipation contributions from each droplet:


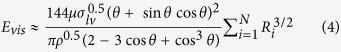


The droplet coalescence must overcome the interfacial adhesion between the droplet and the surface, which is determined by the surface morphology and solid-liquid contact area. The standard expressions for adhesion-induced energy dissipation for the coalescence of multiple Cassie-state droplets (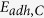) and multiple composite-state droplets (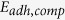) are given below^37^:


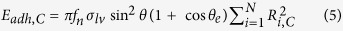



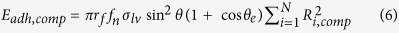


These equations are usually employed to predict the adhesion-induced energy dissipation of the coalescence of two droplets^21^. However, they only consider the contact area underneath the droplets. During the coalescence of groups of droplets, droplets are swept over a larger total solid-liquid contact area than simply their cumulative base contact area with the surface prior to coalescence, resulting in more dissipative surface adhesion. Due to the random starting locations of individual droplets, it is difficult to estimate the specific pathways taken by each droplet across the surface during coalescence in order to describe the exact solid-liquid contact area. We propose a correction factor *λ* to account for the increased effective solid-liquid adhesion contact areas during coalescence as a function of the number and size of multiple droplets, which is expressed as: 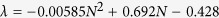 (Supplementary Section 3).

Because the condensate droplets are comparable in size to the microstructures, the line tension (*σ*), is not negligible in the energy analysis^34^,^38^. The line tension-induced energy dissipation at the triple line for the coalescence of multiple Cassie-state droplets (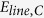) and multiple composite-state droplets (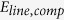), is expressed respectively as:


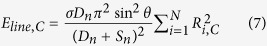



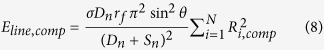


The kinetic energy of the droplet after coalescence must balance the release of surface energy and various dissipation energies:


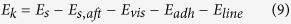


The surface energy of a coalesced droplet of radius *R* is expressed as:


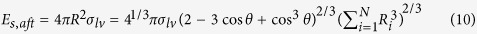


The kinetic energy of the coalesced droplet is given by (based on the mass conservation):


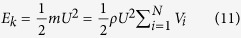


Thus, the droplet jumping velocity is:


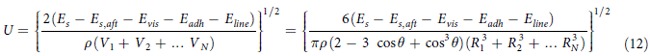


For all of the jumping events shown in Fig. S1e, the surface and dissipation energies are determined from the side-view images and the theoretical jumping velocities are calculated. A line tension *σ* value of 5×10^−8^ J is chosen so as to provide the best agreement with our experimental jumping velocities. The theoretical droplet jumping velocities (closed symbols) are compared to the experimental data in Fig. 6. The theoretical data match very well with the experimental cases where two droplets coalesce; however, when the number of coalescing droplets increases, the theory significantly overpredicts the measured velocities for some cases, specifically when N > 6 (see the last 4 points of the bottom right figure). This may be attributed to the extreme deviations of the droplet jumping trajectories from the surface normal observed with multiple droplet coalescence, in contrast to the near-normal jumping trajectories that follow the coalescence of two droplets. This deviation of the droplet trajectory could be caused by the coalescence of uneven-sized droplets or asymmetric adhesion of the droplets to the microstructures. Once triggered, the coalescence likely occurs in a domino-like fashion, where the initial coalesced droplet sweeps up droplets in its moving path (in contrast to the spontaneous coalescence for pairs of droplets). This deviation of the droplet trajectory is indicative of additional dissipation energies, which may culminate in the formation of a large droplet jumping from the surface with a lowered measured velocity, despite the greater surface energy released upon coalescence. Such additional dissipation energies are not considered in the theoretical predictions (because experimental measurement of the speed of the coalescence process is not feasible by visualization), causing the theory to overpredict the experiments. Although on the hierarchical micro/nano-structured superhydrophobic surface, the jumping velocity for cases with a large number of coalescing droplets is lower compared to two droplet coalescence cases, the multiple droplet series coalescence-induced sweeping removal of neighboring droplets frequently expose fresh spots on the surface for a continual process of nucleation and departure, a potential mechanism to significantly enhance dropwise condensation in a self-sustained manner.

In summary, we investigated the coalescence-induced jumping of condensate droplets from a hierarchical superhydrophobic surface. Unlike prior studies that focused on droplet jumping triggered by the coalescence of two equally sized droplets, our experimental observations indicated that the removal of condensate droplets from the hierarchical surface was dominated by jumping events triggered by the coalescence of more than two droplets. To accurately measure the velocity of droplets that jump in random directions from the surface as a result of multiple-droplet coalescence, and the size differences of all participating droplets, we developed a depth-from-defocus technique to recover the droplet radius and out-of-plane displacement data from single-camera images. Contrary to previous predictions of jumping behaviour, the jumping velocity did not increase with the number of coalescing droplets. We developed a theoretical model that takes into account the coalescing droplet sizes, droplet wetting states, number of coalescing droplets, and the increased surface contact area of groups of coalescing droplets. Our theoretical predictions are consistent with the experimental data for cases where two droplets coalescence; the theory begins to overpredict the measured velocities for cases with a large number of coalescing droplets.

## Methods

### Hierarchical surface fabrication

The hierarchical nanostructured truncated microcone surfaces were fabricated in the Birck Nanotechnology Center at Purdue University. Silicon wafers with a 1 μm-thick SiO_2_ layer were used as the substrates. A photoresist pattern was first created on the silicon wafer using standard photolithography processes to define the microscale cone features. The steps included spinning hexamethyldisilazane (HMDS) and AZ 9260 photoresist at 4000 rpm for 10 s and 30 s, respectively, soft-baking at 100 °C for 5 min, exposing for 55 s at a power of 10 mW/cm^2^ (Karl Suss MJB-3 mask aligner), developing in diluted AZ400K (AZ400K:deionized (DI) water = 1:3) for 4 min, and hard-baking at 90 °C for 30 min. The sample was then immersed in a buffered oxide etch (BOE) solution for 14 min to pattern the SiO_2_, followed by soaking in acetone for 2 min to dissolve the remaining photoresist. The patterned SiO_2_ layer served as the etch mask for the subsequent anisotropic etching. The sample was wet-etched in 25 wt.% tetramethylammonium hydroxide (TMAH) solution at a temperature of 80 °C to achieve an etching rate of ~0.3 μm/min. By controlling the etching time, a silicon surface with truncated microcone features was created after removing the top SiO_2_ etching mask with BOE solution. A metal-assisted chemical etching method was used to fabricate Si nanowires on the micro-structured surface. The surface was first etched with 5% HF aqueous solution for 3 min to produce an H-terminated substrate, and then immersed into an aqueous solution of 4.8M HF and 5 mM AgNO_3_ for 1 min to coat a uniform layer of Ag nanoparticles. The surface was washed with water to remove the extra Ag^+^ ions and then immersed in an etchant composed of 4.8M HF and 0.4M H_2_O_2_. After etching for 3 min in dark conditions (to avoid the influence of photogenerated charges), the wafer was washed repeatedly with water and then immersed in dilute HNO_3_ (HNO_3_:DI water = 1:2) for 30 min to dissolve the Ag catalyst. As a result, the silicon nanowires were grown uniformly across the surface of the microstructures and substrate. To render the surface superhydrophobic, the sample was silanized through immersion in 1 mM *n*-hexane solution of 1H,1H,2H,2H-perfluorooctyl-trichlorosilane for 1 hr, followed by heat treatment at ∼150 °C on a hotplate.

### Side-view observation of condensation dynamics

Experiments were conducted using a custom imaging platform built using a 66 mm-construction optical rail system (Thorlabs). The samples were affixed onto a thermoelectric cooling stage (CP-031, TE Technology, Inc.) using carbon adhesive tape. During testing, the cold plate surface temperature was maintained at 0 °C using a temperature controller (TC-48-20, TE Technology Inc.). Images were obtained using a high-speed camera (FASTCAM 1024 PCI, Photron) operating at 3000 frames/sec with a frame size of 512 × 512 pixels equipped with a wide-range zoom lens at 1000× magnification (VH-Z100R, Keyence). Calibration images yielded an imaging resolution of ~0.8 μm/pixel. A continuous 300 W Xe arc lamp (model 66902, Newport Corp.) was coupled with the lens to provide coaxial illumination in order to increase foreground brightness, and to enhance the visibility of condensate droplets resting on the base as well as the surface microstructures.

Due to the large disparity in droplet size and velocity length scales, it is challenging to capture a high-quality image of a jumping droplet that maximizes pixel resolution and image brightness while reducing image blur. In order to apply depth-from-defocus image processing methods to extract depth-resolved position data, the droplet image must be a high-quality freeze-frame. However, even at the fast recording rate employed (3000 fps), the droplets would travel a significant distance across the image within a single frame, resulting in a streaked image with continuous exposure. A pulsed-LED backlight was incorporated into the experimental setup in order to reduce the effective frame exposure time and generate a still image of the jumping droplet. The LED pulse duration was set to 8 μs during the total frame exposure of 333 μs and was initiated at the start of the frame exposure using a synchronizer (model 505 pulse/delay generator, Berkeley Nucleonics Corp.).

## Additional Information

**How to cite this article**: Chen, X. *et al.* Coalescence-Induced Jumping of Multiple Condensate Droplets on Hierarchical Superhydrophobic Surfaces. *Sci. Rep.* **6**, 18649; doi: 10.1038/srep18649 (2016).

## Supplementary Material

Supplementary Information
