# Supplementary material for: Coalescence-Induced Jumping of Multiple Condensate Droplets on Hierarchical Superhydrophobic Surfaces
Source: Sci Rep. 2016 Jan 4;6:18649. doi: 10.1038/srep18649 (PMC4698694; doi:10.1038/srep18649)
Supplement: Supplementary Information [file srep18649-s1.pdf]

Supporting Information

Coalescence-Induced Jumping of Multiple Condensate Droplets on Hierarchical Superhydrophobic Surfaces

Xuemei Chen, Ravi S. Patel, Justin A. Weibel, and Suresh V. Garimella*

School of Mechanical Engineering and Birck Nanotechnology Center, Purdue University, West Lafayette, Indiana, 47907-2088, USA.

* Correspondence and requests for materials should be addressed to S.V.G (sureshg@purdue.edu)

1. Comparison of the coalesced droplet radii

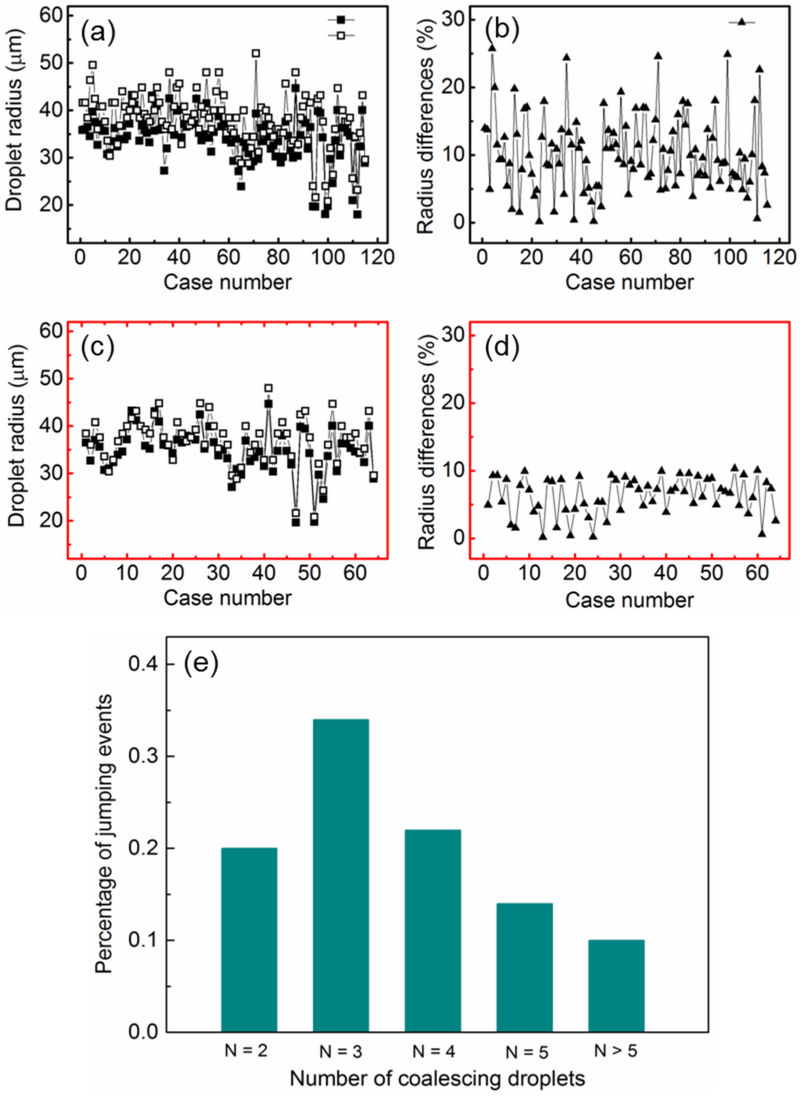

Figure S1. (a-d) Comparison of the measured coalesced droplet radius (open symbols) against the radius of a single droplet containing the volume of all observed coalescing droplets (closed symbols). The radii (left) and absolute difference between the radii (right) is shown for (a, b) all 115 cases acquired and (c, d) after discarding the cases for which the difference is larger than 10%, respectively. The absolute differences between the measured and anticipated droplet radius for all cases vary from 0 to 26%. Of the remaining cases, 50 are selected to achieve (e) a similar distribution of jumping events as viewed normal to the surface (see Figure 4b of the main paper for comparison).

2. Size of initial coalescing droplets

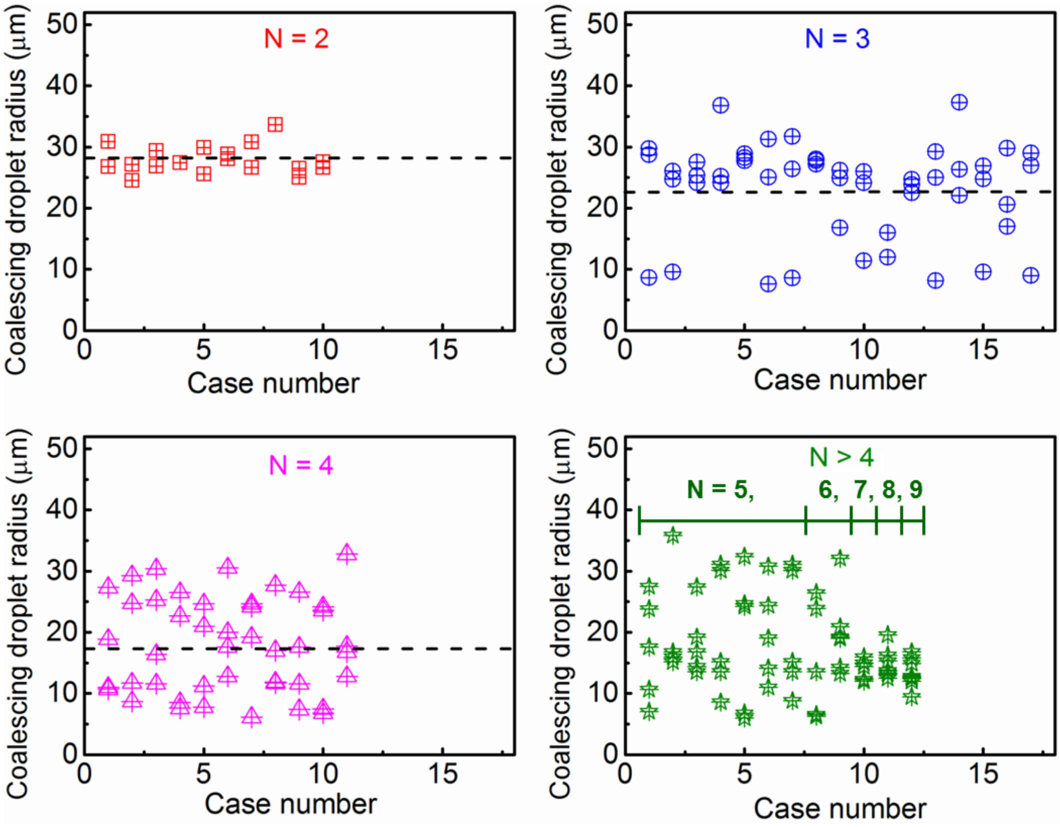

Figure S2. Coalescing droplet radius as a function of the number of coalescing droplets for different cases. The horizontal dashed lines in each figure panel indicate the average droplet radii, which are ~28 μm, ~23 μm, and ~18 μm for N = 2, 3, and 4, respectively.

3. Determination of the correction factor for adhesion-induced energy dissipation during coalescence of multiple droplets

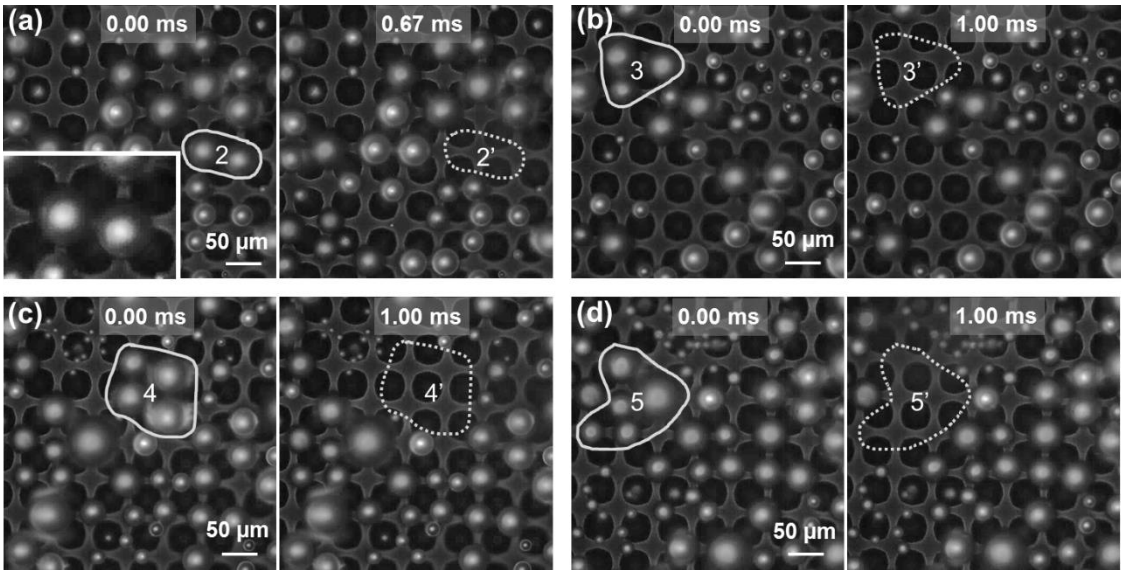

Figure S3. Selected snapshots showing dropwise condensation on the hierarchical superhydrophobic surface. The images on the left show the moment before droplet coalescence (N = 2, 3, 4, and 5) and the images on the right show the moment just after departure, which exposes the substrate (2', 3', 4', and 5'). The inset in (a) shows a close-up image of two coalescing droplets.

The correction factor λ that accounts for the increased effective solid-liquid adhesion contact areas was determined based on the condensation experiments viewing normal to the surface. Figure S3 shows selected dropwise condensation images on the hierarchical superhydrophobic surface. The areas enclosed by solid/dashed lines are denoted as A_{encl}. The left bottom corner of Fig. S3a shows a close-up image of two coalescing droplets. The bases of these two droplets contact four microstructures; the actual contact area of a single droplet with each microstructure sidewall can be approximately as 1/8 of the lateral area of a truncated microcone.

The sample size of the coalescence events is selected based on the distribution of jumping events as shown in Fig. 1e. From each observation of coalescence normal to the surface, we measured the diameters of the initial coalescing droplets (D_{drop}) and surface

areas contained within a perimeter drawn around the group of coalescing droplets (A_{encl}), and calculated the contact area between the droplets and the microstructure sidewalls (A_{side}). Due to the different sizes of the droplets for each coalescence event, the total area ($A_{cont} + A_{encl}$) is normalized by the average size of the initial coalescing droplets as: $(A_{cont} + A_{encl})/D_{drop}$. After averaging this quantity for each bin of the same number of coalescing droplets (*N*), it is denoted as α_k (where k = 2 to *N*). Considering that the two-droplet coalescence case does not require any correction to the adhesion-induced energy dissipation, the value α_2 is used as a baseline to evaluate the relative increase in the solid-liquid contact area as the number of coalescing droplets increases past two. The ratio of α_k/α_2 is denoted as λ_k (k = 2 to *N*), the correction factor. By a polynomial fitting of λ_k, we obtained an expression for the correction factor as a function of the number of coalescing droplets, given as: $\lambda = -0.00585N^2 + 0.692N - 0.428$.
